# Detection Methods for Multi-Modal Inertial Gas Sensors

**DOI:** 10.3390/s22249688

**Published:** 2022-12-10

**Authors:** Fehmi Najar, Mehdi Ghommem, Samed Kocer, Alaa Elhady, Eihab M. Abdel-Rahman

**Affiliations:** 1 Department of Mechanical Engineering, College of Engineering at Al Kharj, Prince Sattam bin Abdulaziz University, Al-Kharj 11942, Saudi Arabia; 2 Applied Mechanics and Systems Research Laboratory (LR03ES06), Tunisia Polytechnic School, University of Carthage, Tunis 1054, Tunisia; 3 Department of Mechanical Engineering, American University of Sharjah, Sharjah P.O. Box 26666, United Arab Emirates; 4 Systems Design Engineering, University of Waterloo, Waterloo, ON N2L 3G1, Canada

**Keywords:** arch beam, asymmetric actuation, gas sensors, bifurcation-based detection, modal ratio, differential capacitance

## Abstract

We investigate the rich potential of the multi-modal motions of electrostatically actuated asymmetric arch microbeams to design higher sensitivity and signal-to-noise ratio (SNR) inertial gas sensors. The sensors are made of fixed–fixed microbeams with an actuation electrode extending over one-half of the beam span in order to maximize the actuation of asymmetry. A nonlinear dynamic reduced-order model of the sensor is first developed and validated. It is then deployed to investigate the design of sensors that exploit the spatially complex and dynamically rich motions that arise due to veering and modal hybridization between the first symmetric and the first anti-symmetric modes of the beam. Specifically, we compare among the performance of four sensors implemented on a common platform using four detection mechanisms: classical frequency shift, conventional bifurcation, modal ratio, and differential capacitance. We find that frequency shift and conventional bifurcation sensors have comparable sensitivities. On the other hand, modal interactions within the veering range and modal hybridization beyond it offer opportunities for enhancing the sensitivity and SNR of bifurcation-based sensors. One method to achieve that is to use the modal ratio between the capacitances attributed to the symmetric and asymmetric modes as a detector, which increases the detection signal by three orders of magnitude compared to a conventional bifurcation sensor. We also present a novel sensing mechanism that exploits a rigid arm extending transversely from the arch beam mid-point and placed at equal distances between two side electrodes. It uses the asymmetry of the arch beam motions to induce rotary motions and realize a differential sensor. It is found to increase the detection signal by two orders of magnitude compared to a conventional bifurcation sensor.

## 1. Introduction

Micro-electro-mechanical systems (MEMS) based devices have demonstrated great capability in sensing applications, such as bio-mass [1,2,3], gas [4,5,6,7,8], chemical [9,10], and pressure sensors as well as [11,12,13,14,15] inertial measurements [16,17,18]. MEMS technology is an attractive alternative for such applications, as it offers a viable platform made of microstructures and on-chip electronics that can simultaneously deliver low system costs, reliability, integration with electronics, and ease of use. In particular, MEMS gas sensors rely on the conversion of the presence or concentration of a target gas into a resolvable signal via transduction techniques [4]. Such sensors can fulfill an emerging need for the real-time detection and monitoring of toxic gas leaks, environmentally hazardous gases, and other environmental monitoring tasks.

Several detection mechanisms have been proposed to enhance the performance of electrostatic MEMS mass and gas sensors, including the activation of more sensitive higher-order modes via partial-length electrode actuation [7,19,20,21], operation near bifurcation points to exploit abrupt jumps to a larger-amplitude response upon detection [1,5,6,10,19,22,23,24], mode localization [25,26,27,28,29], and buckling and snap-through of bistable structures [21]. Jaber et al. [19] demonstrated that the second symmetric mode of a microbeam, actuated via an electrode covering two thirds of its length, is more sensitive than the first symmetric mode. They also showed [7,20] that the first anti-symmetric mode of the microbeam, actuated via a half-length electrode, is more sensitive than the first symmetric mode. Hajjaj et al. [21] showed the possible exploitation of the multi-modal excitation of electrothermally buckled microbeams for gas sensing.

Given the rich dynamics of electrostatically actuated arch microbeams, several studies have examined their potential use in sensing and actuation [15,24,30,31,32,33,34]. For example, Najar et al. [24] demonstrated the potential of electrostatic initially curved microbeams to serve as bifurcation gas sensors in either binary mode by monitoring for an abrupt transition from regular periodic motions to chaotic motions upon gas detection, or analog mode, by relating the measured RMS of the response phase angle to the gas concentration.

Arch beams expand the range of stable motions under electrostatic actuation. This may be prove useful in sensing since it allows for the creation of stronger signals and potentially higher signal-to-noise ratios (SNR). Arch beams also give rise to crossing and veering phenomena between the symmetric and asymmetric modes, as the natural frequencies of symmetric modes increase with the initial rise while those of the asymmetric modes remain unaffected. While symmetric mechanical and electrical energy distributions across the sensor result in crossing between those modes, asymmetry results in veering between the modes. It can be introduced to the electrical field using partial electrodes with the limiting case being a half-length electrode. In this paper, we examine potential uses of half-length electrode arch microbeams in inertial gas sensing. First, we conduct a comprehensive nonlinear analysis of the sensor dynamics to examine the interactions between its symmetric and asymmetric modes. Based on that, we conduct a comparison among two traditional detection mechanisms and two novel mechanisms that exploit modal interactions between the first symmetric and the first anti-symmetric beam modes. The present work constitutes the first stage in a multi-phase multidisciplinary research program meant to investigate the feasibility of those novel gas detection mechanisms.

## 2. Mathematical Model of Gas Sensor

**Figure 1:**
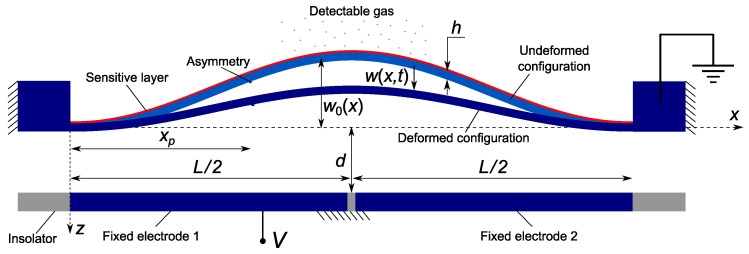
Schematic of the asymmetric arch beam gas sensor.

The gas sensor under study, Figure 1, is made of a clamped–clamped shallow arch of length *l* and width *b*, and thickness *h*. It is electrically actuated by a voltage drop applied between the beam and a side electrode. Different actuation configurations are considered to enable the activation of higher symmetric and anti-symmetric vibration modes. For instance, asymmetric actuation via electrode 1 or 2, so-called ‘half’ electrode configuration), enables the activation of the first anti-symmetric mode, whereas symmetric actuation via both electrodes would not be able to activate that mode. We note that the dynamic response of straight and arch microbeams under partial electrode actuation has been extensively studied in the literature [7,19,20,31,32,35,36]. Our objective is to demonstrate the exploitation of their dynamic features to develop novel and efficient detection gas mechanisms.

The capacitance per unit length between the arch microbeam and the fixed half-length electrode can be written, including the fringing field, as [37]

(1) $$\begin{array}{cc} C_{l} & =H_{c}\left(\hat{x}\right)\left(\frac{\varepsilon b}{d-\hat{w}+{\hat{w}}^{0}}-0.65\varepsilon \log\left(d-\hat{w}+{\hat{w}}^{0}\right)\right) \end{array}$$

where *d* is the initial gap distance between the line connecting the fixed supports and the electrode, ${\hat{w}}^{0}$ is the initial rise at the mid span. The resulting electrostatic force per unit length acting on the beam is given by

(2) $$\begin{array}{cc} F_{e} & =\frac{1}{2}\frac{\partial C_{l}}{\partial \hat{w}}V^{2}\\ \; & =\frac{1}{2}V^{2}H_{c}\left(\hat{x}\right)\frac{\varepsilon b}{{\left(d-\hat{w}+{\hat{w}}^{0}\right)}^{2}}\left(1+\frac{0.65}{b}\left(d-\hat{w}+{\hat{w}}^{0}\right)\right) \end{array}$$

Following Euler–Bernoulli beam assumptions, accounting for the mid-plane stretching and squeeze-film damping, and electrostatic force in Equation (2), the governing equation of the deflection of the microbeam can be written in nondimensional form as [15]

(3) $$\begin{array}{cc} \; & \left(1+\delta m\right)\ddot{w}+\left(\alpha_{c}+\frac{\alpha_{\mu }}{{\left(1-w+w^{0}\right)}^{3}}\right)\dot{w}+\left(1+\alpha_{1}\delta \left(x_{p}\right)\right)w^{iv}\\ \; & =\alpha_{2}\left(w^{\prime \prime }-{w^{0}}^{\prime \prime }\right)\int_{0}^{1}\left({\left(w^{\prime }\right)}^{2}-2w^{\prime }{w^{0}}^{\prime }\right)dx+\alpha_{3}V^{2}H_{c}\left(x\right)\frac{1+\gamma (1-w+w^{0})}{{\left(1-w+w^{0}\right)}^{2}} \end{array}$$

subject to the boundary conditions

(4) $$\begin{array}{cc} \; & w\left(0,t\right)=0\;w^{\prime }\left(0,t\right)=0\\ \; & w\left(1,t\right)=0\;w^{\prime }\left(1,t\right)=0 \end{array}$$

where primes denote derivative with respect to the nondimensional parameter *x* and over dots denote derivative with respect to the nondimensional time parameter *t*.

The function $H_{c}$ is defined for each of the excitation cases by

(5) $$H_{c}\left(x\right)=\left\{\begin{array}{cc} H\left(x\right)-H\left(x-\frac{1}{2}\right) & for\;half-electrode\;actuation\\ 1 & for\;full-electrode\;actuation \end{array}\right.$$

where H(x) is the Heaviside function.

The nondimensional variables, axial position, transverse deflection, initial rise function and time, are respectively obtained by letting

(6) $$\begin{array}{c} \hat{x}=xL,\;\hat{w}=wd,\;{\hat{w}}^{0}=w^{0}d,\;\hat{t}=t\tau \end{array}$$

where $\tau =\sqrt{\frac{mL^{4}}{EI}}$, m=ρbh, *E* is Young’s modulus, $I=\frac{bh^{3}}{12}$, *b* and *h* are the width and thickness of the microbeam, and ρ is its density.

The ratio of the mass added by gas sorption to the beam mass is denoted δm. A small but finite perturbation of the microbeam section rigidity $\alpha_{1}$ is placed at $x_{p}=0.97$, as shown in Figure 1, to account for naturally occurring asymmetries in the microbeam dimensions and material. The initial deflection of the microbeam is described by

(7) $$w^{0}\left(x\right)=\frac{w_{o}}{2}\left(1-\cos\left(2\pi x\right)\right)$$

where $w_{o}$ is the initial rise at mid span. The actuation voltage is defined as

$$V\left(t\right)=V_{DC}+V_{AC}\cos\left(\Omega t\right)$$

where $V_{DC}$ is the bias voltage and $V_{AC}$ and Ω are the amplitude and frequency of harmonic excitation.

The nondimensional coefficients in Equation (3) are given by

(8) $$\begin{array}{cc} \alpha_{2}=6\frac{d^{2}}{h^{2}},\;\alpha_{3}=\frac{\varepsilon bL^{4}}{2d^{3}EI},\; & \alpha_{c}=\frac{cL^{4}}{EI\tau },\;\alpha_{\mu }=\frac{\mu b^{3}}{1+6.8636K_{n}^{0.9906}}\frac{L^{4}}{\tau EId^{3}}\\ \; & K_{n}=\frac{\lambda_{o}p_{o}}{p_{a}d},\;\gamma =0.65\frac{d}{b} \end{array}$$

where the $\epsilon =8.85\times 10^{-12}$ C${}^{2}$N${}^{-1}$m${}^{-2}$ is permittivity of the dielectric medium, *c* is the linear viscous damping coefficient, μ is the dynamic viscosity of air at room temperature and atmospheric pressure $p_{o}=101.325$ kPa, $p_{a}$ is the ambient pressure, $K_{n}$ is the Knudsen number and $\lambda_{o}=65$ nm is the mean free path.

## 3. Model Validation

### 3.1. Experimental Setup and Results

**Figure 2:**
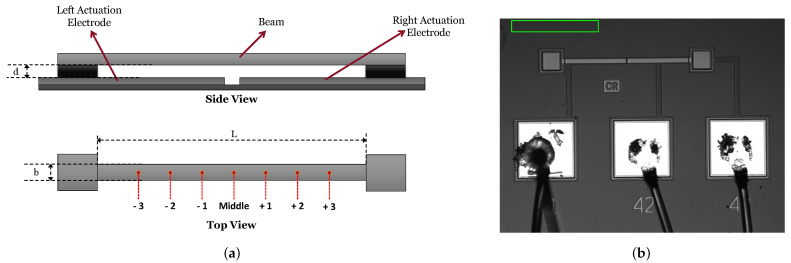
MEMS device under test: (**a**) side and top views showing the locations of the measurement points along the beam span, (**b**) a microscopic image.

The model predictions are validated experimentally. Specifically, the experimental study is carried out to investigate the impact of half-length electrode actuation on the response of the sensor, and whether it can activate interactions between symmetric and asymmetric modes. These interactions are the focus of this work and proposed sensing mechanisms. Schematics of the fixed-fixed beam under test, showing the side and top views of the device, are shown in Figure 2a. The device was fabricated in the PolyMUMPs process, and the beam was patterned in the POLY2 layer with a thickness of h=2 µm. Two identical left and right half-length electrodes were patterned below the beam, in the POLY0 layer, with a capacitive gap of d=2 µm. The length and width of the beam are L=400 µm and b=15 µm. Figure 2b shows a microscopic top view image of the device-under-test.

**Figure 3:**
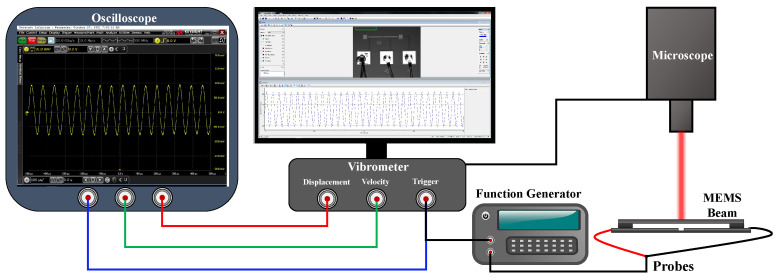
A schematic of the experimental setup.

The experimental setup in Figure 3 was used to measure the device response using the Laser Doppler Vibrometer, LDV. The beam was actuated using a sinusoidal voltage from the B&K Precision-4054 function generator. The electrostatic force on the beam under a single-tone (pure AC) voltage occurs at twice the applied frequency. Therefore the beam was actuated in the vicinity of its natural frequency using a single-tone sinusoidal voltage near half the said frequency.

During half-electrode excitation, we actuate the left electrode at the natural frequency of the beam’s nth mode while grounding the right electrode and the beam. During full-electrode excitation, both bottom electrodes are actuated while the beam is grounded. In both case, the excitation amplitude is $V_{AC}=2$ V and the bias is $V_{DC}=2$ V.

**Figure 4:**
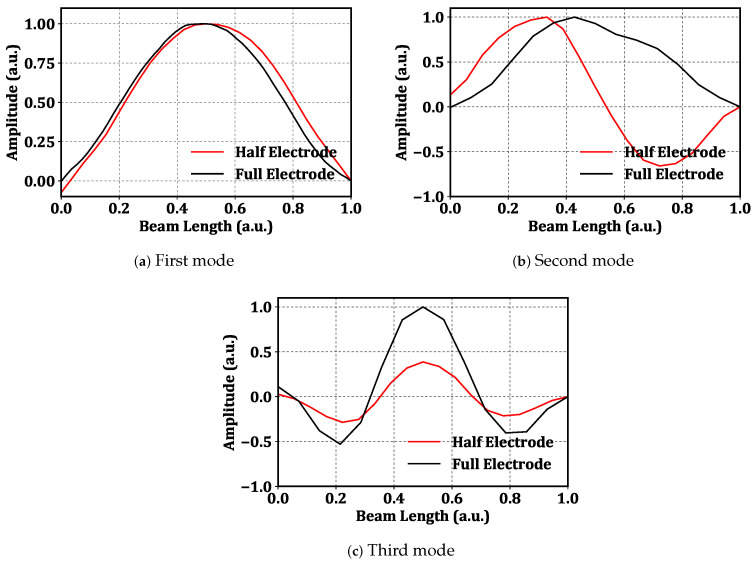
The first three mode shapes of the beam under half-electrode excitation (red) and full-electrode excitation (black) with the voltage waveform $V_{AC}=2$ V and $V_{DC}=2$ V.

The LDV was used to scan the beam at a grid made of 50 equidistant and record the magnitude and phase of the velocity at each points. The data are used to construct the mode shape of the vibration. Unlike several previous works, the analysis of the mode shapes was carried out experimentally. For instance, Alcheikh et al. [36] presented an experimental study on clamped–clamped microbeams actuated via different electrode configurations: full, half, and two third. The mode shapes were obtained using a finite element analysis model. Figure 4 shows the first three mode shapes of the beam under half-electrode excitation (red line) and full-electrode excitation (black line). The figure shows that the half-electrode excitation suppresses the left half of the mode shape by a few percentage points.

The first and third modes of the beam are symmetric, while the second mode is antisymmetric. Symmetric modes can be obtained by either half-electrode or full-electrode excitation; Figure 4a,c. However, Figure 4b shows that realizing an antisymmetric mode requires antisymmetric excitation. Therefore, under full-electrode excitation, the second mode cannot be directly excited, and the beam responds with first mode-like forced response, even when excited at the second mode’s natural frequency due to the fact that the excitation force has negligible projection on the second mode; Figure 4b.

We swept the excitation frequency in the vicinity of each mode’s natural frequency, over a duration of 20 ms using the voltage waveform $V_{AC}=2$ V and $V_{DC}=2$ V. The velocity was captured in the time domain at the key points along the beam labeled in Figure 2. The captured time-domain data were post-processed to obtain the frequency response, the RMS velocity over a two-period time window, as the frequency of excitation was swept up.

**Figure 5:**
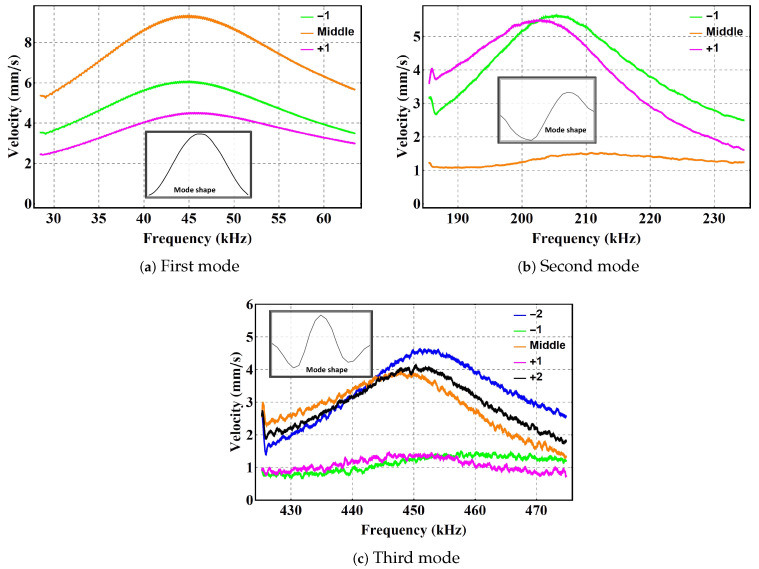
The frequency–response curves at key points along the curved beam in the vicinity of the first, second, and third natural frequencies under half-electrode excitation with the voltage waveform $V_{AC}=2$ V and $V_{DC}=2$ V.

Figure 5 shows the frequency response in the vicinity of the natural frequency of the lowest three modes. The figure shows the RMS velocity at key positions along the beam under half-electrode excitation. The excitation frequency was swept from 25 kHz to 65 kHz, 185 kHz to 235 kHz, and 425 kHz to 475 kHz to capture those responses. Figure 5a shows that point −1 (green line) to the left of the mid-point moves 33% more than point +1 (magenta line) at the same distance to the right of the mid-point. Thus, the first mode is not perfectly symmetric due to the asymmetric nature of half-electrode actuation. The same can be observed in Figure 5c for the third mode.

### 3.2. Comparative Study

**Figure 6:**
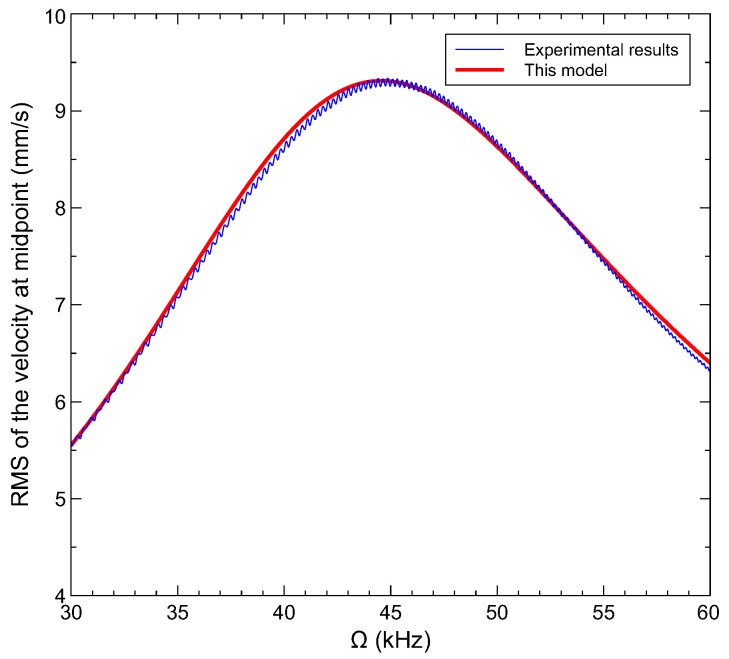
Frequency responses of the microbeam under half electode length actuation: model vs. experiments.

We develop a nonlinear reduced-order model following the numerical procedure described in [38] to obtain the dynamic response of the gas sensor. The mode shapes of a straight clamped–clamped microbeam are used in a Galerkin expansion in order to discretize the equation of motion and obtain a set of ordinary differential equations (ODEs). The formulation of the obtained dynamic reduced-order model is shown in the appendix. The ODEs are solve using the finite difference method and an arc-length continuation technique [24,39,40]. In Figure 6, we compare the simulated frequency response obtained using the proposed model with that measured experimentally for a straight microbeam ($w_{o}=0$ µm) actuated via a half-electrode. The comparative study reveals a good agreement between the numerical and the experimental results. We note that effective microbeam parameters used in the simulations are listed in Table 1. The first three natural frequencies obtained from the present model compare well to those measured experimentally, as shown in Table 2.

## 4. Model Results

With a view to using the model to investigate the feasibility and potential advantages of novel detection mechanisms, we start by deploying it to establish the basic response of the curved beam resonator. We consider a gas sensor made of an arch beam under electric actuation. Its specifications are given in Table 3. We use the developed model to examine the static and dynamic responses of the gas sensor. We investigate the potential of novel detection mechanisms that exploit the rich dynamics of arch beams.

### 4.1. Static Response

**Figure 7:**
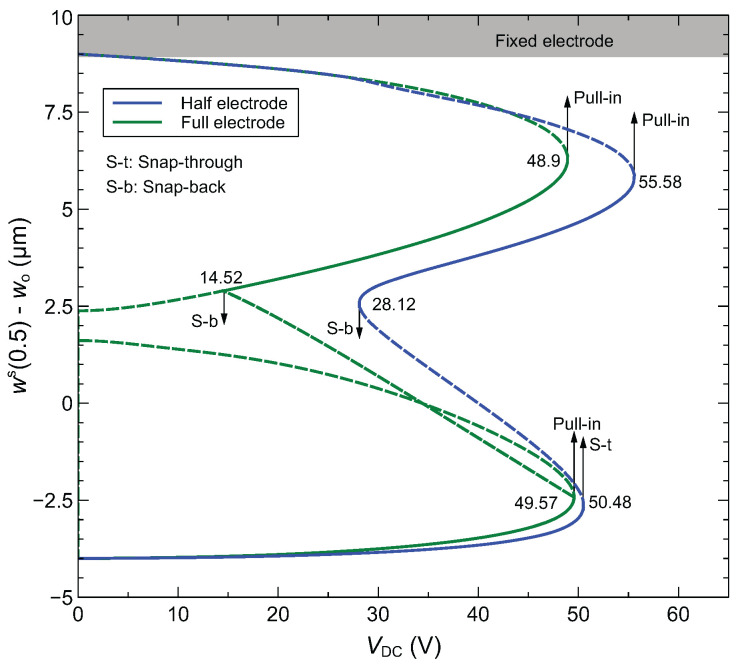
Static response at the midpoint of the arched microbeam: full vs. half-electrode actuation. The initial rise is set to $w_{o}$ = 4 μm.

We first examine the static response of the arched microbeam for the full- and half-electrode actuation configurations. The formulation of the static reduced-order model using the differential quadrature method (DQM) is given in Appendix A. We show in Figure 7 the static response obtained at the midpoint for different applied DC voltages. These results demonstrate the significant qualitative and quantitative changes in the nonlinear static response when shifting from full-electrode to half-electrode actuation. In fact, only the lower stable branches obtained for low DC voltages and the higher unstable branches near touch down overlap. The bifurcation points leading to pull-in, snap-through and snap-back behaviors vary noticeably between the two actuation configurations. Furthermore, separate unstable branches are observed near the snap-back point of the full-electrode configuration while an unstable branch corresponding to an asymmetric response of the microbeam is only observed for this configuration [24]. The half-electrode design is highly asymmetric, which eliminates the occurrence of symmetry breaking bifurcation.

**Figure 8:**
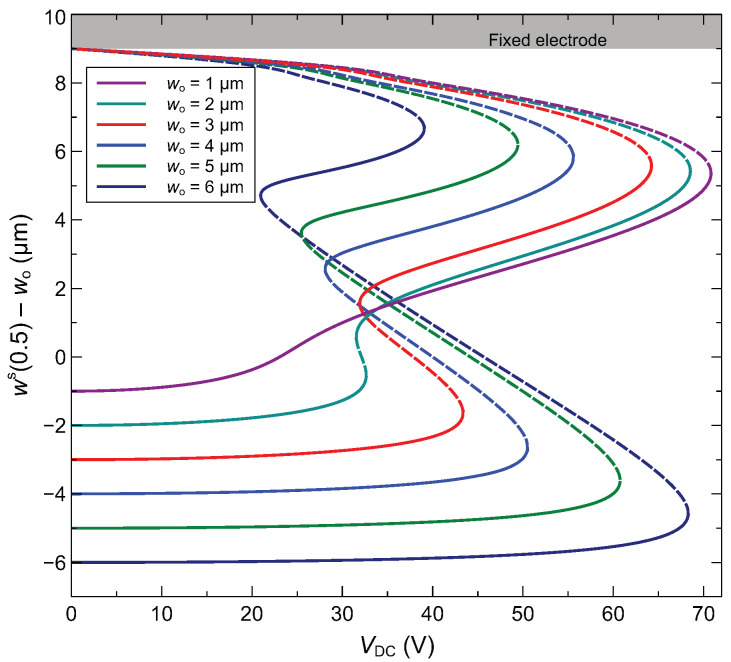
Static response at the midpoint of the arch microbeam for different initial rises (half-electrode actuation).

In the subsequent analysis, the focus will be on half-electrode actuation to exploit symmetry switching for sensing purposes. The initial rise of the arched microbeam $w_{o}$ constitutes an important design parameter. This can be controlled by applying a constant axial stress via an electrothermal process based on the Joule effect. In Figure 8, we plot the static response of the arched microbeam for varying initial rise. Both stable and unstable branches of the response are shown. It is noticed that the different bifurcation points are monotonically sweeping their positions with respect to the applied DC voltage such that the lower bifurcation point is shifting to higher voltage and the upper one is shifting to lower voltages. As a result, for high values of the initial rise (above 5 μm), the upper and lower bifurcation points correspond to snap-through and pull-in, respectively. As for lower values of initial rise, the upper bifurcation point corresponds to a pull-in point, and the lower one to a snap-through point. For all initial rises, the middle bifurcation point is the snap-back point.

### 4.2. Modal Analysis

In this section, we conduct a free vibrations analysis of arch microbeams by solving the corresponding linear damped eigenvalue problem, Equation (3), using the differential quadrature method (DQM) [24]. The formulation of the eigenvalue problem is shown in Appendix B. The number of DQM points is set to n=21 to guarantee convergence of the numerical solutions. Taking into account the damping and applied DC voltage, we calculate the natural frequencies and the corresponding mode shapes of arch microbeams. The resulting modes are dubbed in terms of their initial configuration in the absence of initial rise and voltage as mode 1, the first symmetric mode; mode 2, the first asymmetric mode; and mode 3, the second symmetric mode.

**Figure 9:**
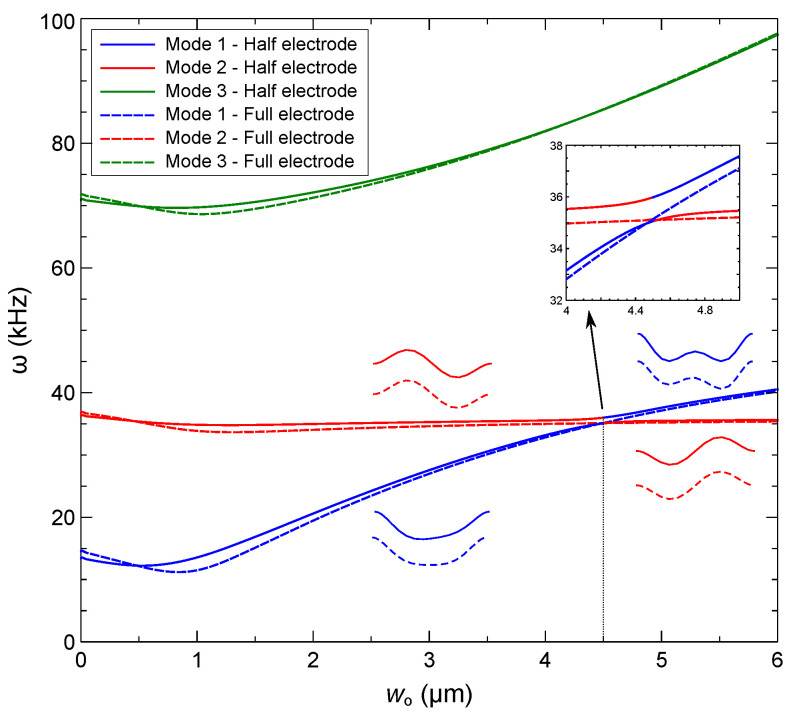
Variation of the first three modes’ natural frequencies with the initial rise $w_{o}$ under a DC voltage of $V_{DC}=15$ V applied across the half-electrode (solid lines) and the full-electrode (dashed lines).

**Figure 10:**
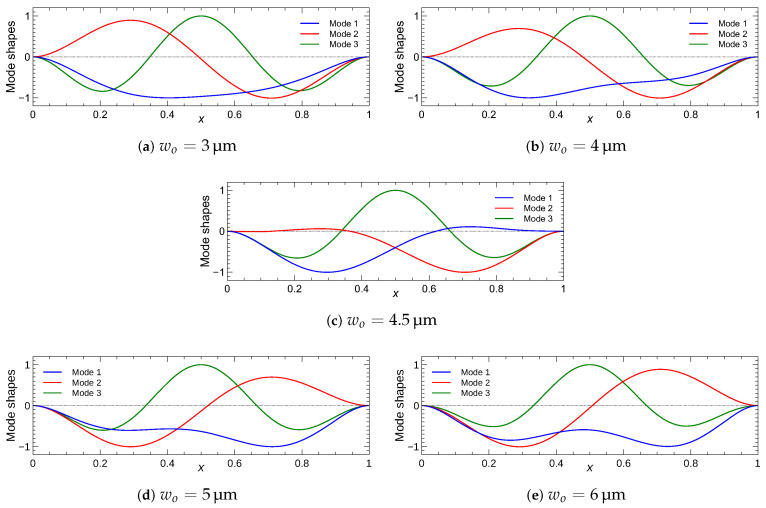
Shapes of the first three modes subject to a DC voltage of $V_{DC}=15$ V across a half-electrode for five values of the initial rise $w_{\circ }$.

We plot in Figure 9 variations in the natural frequencies of the first three modes as functions of the initial rise $w_{o}$ under a DC voltage of $V_{DC}=15$ V. We note that Mode 1 and Mode 2 refer to the first symmetric and first asymmetric mode shapes rather than the numerical value of their natural frequencies. The crossing between the first two natural frequencies, observed in the full-electrode case, changes into veering in the half-electrode case. Veering is observed near $w_{o}=4.5$ µm, where the natural frequency of mode 1 approaches that of mode 2. These modes are similar in shape but out of phase, as can be seen in Figure 10c. This is an indicator of a strong interaction between the two modes. The natural frequencies for the selected values of the initial rise $w_{o}$ around the veering point are listed in Table 4. Since the veering point varies with static transverse loads, the DC voltage (or RMS of the voltage waveform) was held constant at 15 V throughout the present numerical study. Beyond the crossing or veering point, the first symmetric mode is hybridized, as illustrated in Figure 9. The impact of the initial rise $w_{\circ }$ on the natural frequency of asymmetric mode (Mode 2) is insignificant throughout the range of interest. On the other hand, the natural frequency of the first symmetric mode (Mode 1) increases with the initial rise.

For comparison, we also show in Figure 9 the natural frequencies of the first three modes under full-electrode actuation with $V_{DC}=15$ V. While the natural frequencies and mode shapes are similar to those of the half-electrode case, the symmetry of the electrical and mechanical domains result in crossing of modes 1 and 2 rather than the veering seen under asymmetry of the electrical field in the former case [41].

We used the model to investigate the interaction between the mode shapes around the veering point under half-electrode actuation. Figure 10, shows the first three mode shapes for five selected values of the initial rise under a DC voltage of $V_{DC}=15$ V. We note that mode 1 and mode 2 refer to the first symmetric and first asymmetric modes, respectively. The shape of mode 2 remains unchanged except at the veering point, where the two modes are symmetric with respect to the mid span point. On the other hand, mode 1 hybridizes gradually with mode 2 as it approaches and crosses the veering point [31,41]. It is important to note here that the veering and the subsequent hybridization phenomena are occurring in a continuous manner with a smoothly increasing significance before the veering point, and then their effect decreases as the initial rise is further increased. In the present study, our main focus is on the half electrode design. We examine the strength of the interaction between the mode shapes, which is controlled by the initial rise of the arch beam. The shape of mode 3 remains unchanged throughout this process.

### 4.3. Dynamic Analysis of Arch Microbeams

In this section, we obtain the frequency–response curves of half-electrode actuated arch microbeams. The response orbits are evaluated following the numerical procedure described in [38]. The mode shapes of a straight clamped–clamped microbeam are used in a Galerkin expansion to discretize the equation of motion and obtain a set of ordinary differential equations (ODEs). The resulting dynamic reduced-order model is given in Appendix C. The ODEs are numerically solved using the finite difference method and an arc-length continuation technique [24,39,40].

**Figure 11:**
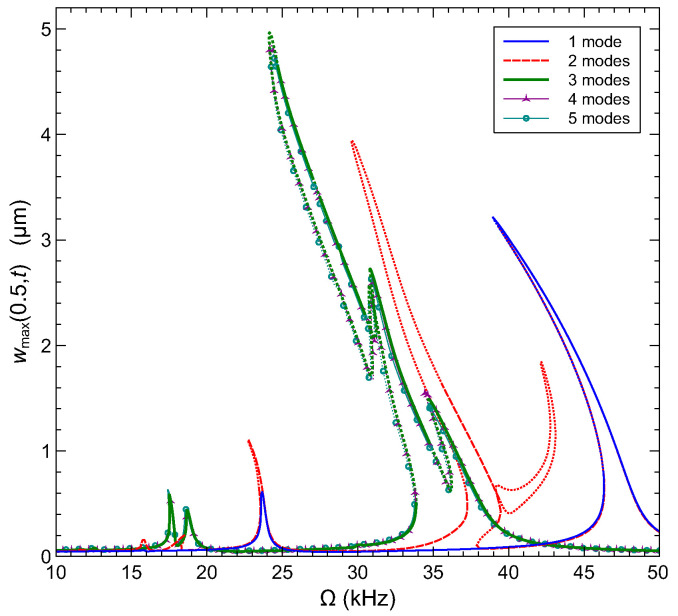
The frequency–response curves obtained using 1–5 mode Galerkin expansions for an arch microbeam with $w_{o}=5$ µm excited by a half-electrode with the voltage waveform $V_{DC}=V_{AC}=12.2$ V. Solid and dashed lines denote stable orbits. Dotted lines denote unstable orbits.

We first determine the number of modes required for the convergence of the dynamic solution. In Figure 11, we compare the frequency–response curves obtained using 1–5 mode Galerkin expansions for an arch microbeam with an initial rise of $w_{o}=5$ µm in the frequency range [10−50] kHz. The curves describe the maximum deflection over the orbit of the mid-point with respect to a line connecting the supports $w_{\max}\left(0.5,t\right)-w_{\circ }$. The microbeam is excited by a the voltage waveform with $V_{DC}=V_{AC}=12.2$ V. The RMS of this waveform is equivalent to $V_{\mathrm{RMS}}=15$ V resulting in natural frequencies identical to those shown in Figure 9 and Table 4. Under these conditions, the natural frequencies of modes 1 and 2 appear in this range and are closely spaced. As a result, modal interactions between their primary resonances are expected. This behavior is recovered using three or more modes expansions as shown in Figure 11. The one and two mode expansions are qualitatively incorrect. On the other hand, the addition of the fourth and fifth modes does not seem to significantly improve the model accuracy. Therefore, we adopt a three-mode approximation for the rest of this paper. In this respect, the developed model is consistent with the literature on the required number of modes to achieve convergence [35].

**Figure 12:**
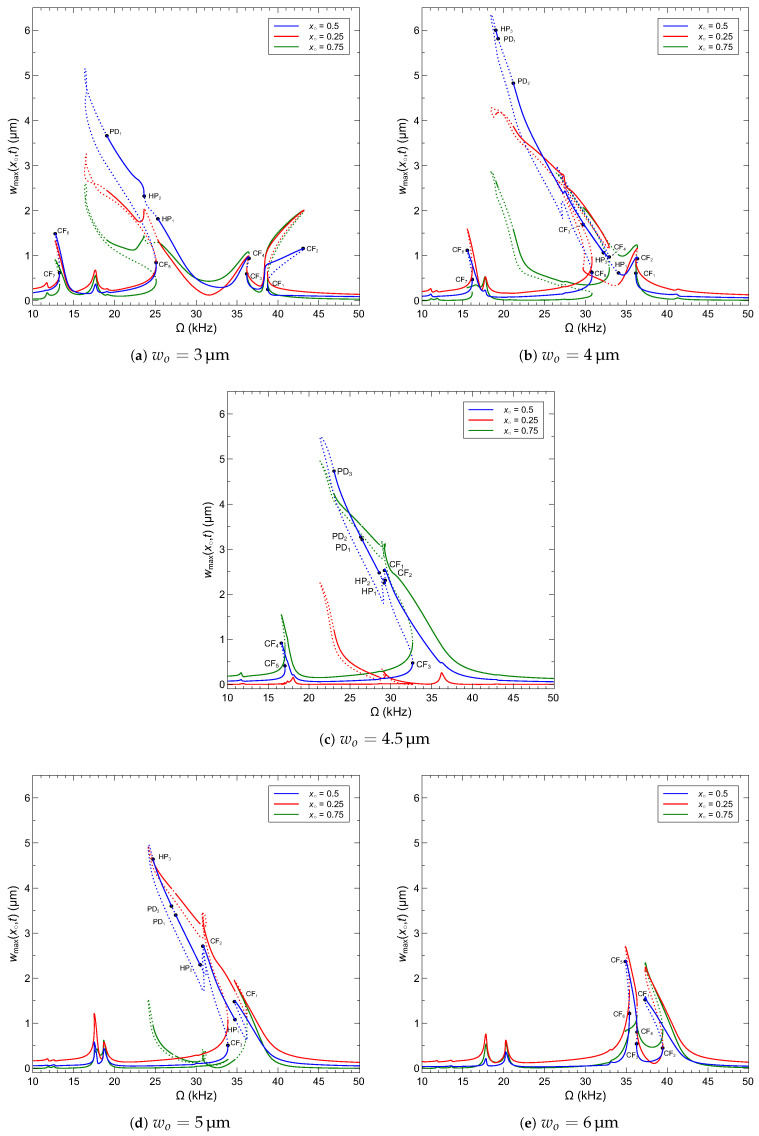
The frequency-response curves of an arch microbeam with an initial rise of (**a**) $w_{o}=3$ µm, (**b**) $w_{o}=4$ µm, (**c**) $w_{o}=4.5$ µm, (**d**) $w_{o}=5$ µm and (**e**) $w_{o}=6$ µm excited by a half-electrode with the voltage waveform $V_{DC}=V_{AC}=12.2$ V. Stable orbits are marked by solid lines. Unstable orbits are marked by dashed lines.

We show in Figure 12 the frequency response of the arch microbeam for five values of the initial rise. $w_{o}=3$, 4, 4.5, 5 and 6 µm, under the voltage waveform $V_{DC}=V_{AC}=12.2$ V. The figures show the maximum displacement realized by the microbeams’s quarter-point $w_{\max}\left(0.25,t\right)$, mid-point $w_{\max}\left(0.5,t\right)$, and three quarters-point $w_{\max}\left(0.75,t\right)$ over the orbit. The stability of each orbit is determined by computing its Floquet multipliers. Stable orbits are marked by solid lines and unstable orbits are marked by dashed lines The location of and type of bifurcation are determined by observing changes in the number and/or stability of orbits available over the frequency spectrum. In all cases, the asymmetric actuation via the half-electrode results in excitation of both the symmetric and asymmetric modes (modes 1 and 2).

For the low initial rise case of $w_{o}=3$, distinct primary resonances and the superharmonic resonances of order 2 of modes 1 and 2 can be identified near their two natural frequencies and half of their natural frequencies in Figure 12a. The superharmonic resonance of order 2 of mode 3 is also observed near 38 kHz. While these resonances can be seen along the beam span, the relative magnitudes vary depending on the observation point. In the primary resonance of mode 1, for example, the response of the mid-point is larger than that of the quarter-point which is larger than that of the three quarters-point $w_{\max}\left(0.5,t\right)>w_{\max}\left(0.25,t\right)>w_{\max}\left(0.75,t\right)$. On the other hand, the response of the three quarters-point is slightly larger than that of the quarter-point and mid point in primary resonance of mode 2 $w_{\max}\left(0.75,t\right)>w_{\max}\left(0.25,t\right)\;\&\;w_{\max}\left(0.5,t\right)$. Bifurcation analysis identified seven cyclic-fold (CF_i_) bifurcations, where a Floquet multiplier exits the unit circle through +1; two Hopf bifurcations (HP_i_), where two complex conjugate multipliers exit the unit circle; and a period-doubling (PD) bifurcation, where a multiplier exits the unit circle through −1. The bifurcation points are marked accordingly on the mid-point curve. While the response of mode 1 shows evidence of softening, the response of modes 2 and 3 show evidence of hardening.

The primary and superharmonic resonances of modes 1 and 2 start to merge as the initial rise increases to $w_{o}=4$ µm, Figure 12b and the natural frequency of mode 1 approaches that of mode 2. The stronger interaction between the modes results in more elaborate dynamics with three additional cyclic-fold bifurcations CF_i_, a third Hopf bifurcation HP_3_, and second (reverse) period-doubling bifurcation PD_2_ appearing on the merged primary resonance curve. The responses of the quarter-point and mid-point are similar and larger than that of the three quarters-point $w_{\max}\left(0.5,t\right)\;\&\;w_{\max}\left(0.25,t\right)>w_{\max}\left(0.75,t\right)$. Additionally, we observe the fading of the superharmonic of order 2 of mode 3.

The merger between modes 1 and 2 is complete at the veering point $w_{o}=4.5$ µm, where their natural frequencies are almost identical, Table 4, and their mode shapes are anti-symmetric, Figure 10c. In this case, their primary and superharmonic resonances are almost indistinguishable; Figure 12c. The response along the beam span undergoes a reversal as the response at the three quarters-point and mid-point dominate that at the quarter-point $w_{\max}\left(0.75,t\right)\;\&\;w_{\max}\left(0.5,t\right)>w_{\max}\left(0.25,t\right)$ indicating a shift in the phase relationship between the symmetric and asymmetric modes.

Beyond the veering point. $w_{o}=5$ µm, the modes assume distinct shapes, Figure 10d, and their natural frequencies start separating as that natural frequency of mode 1 continues to increase beyond that of mode 2, Table 4. While their primary and superharmonic resonances show evidence of strong interaction, their peaks start to pull-off from each other, Figure 12d. However, the order of the response along the beam span is maintained with the response at the three quarters-point and mid-point continuing to dominate that at the quarter-point $w_{\max}\left(0.75,t\right)\;\&\;w_{\max}\left(0.5,t\right)>w_{\max}\left(0.25,t\right)$.

As the natural frequency of mode 1 continues to increase beyond that of mode 2 with the initial rise, $w_{o}=6$ µm, the primary and superharmonic resonances of the two modes become completely distinct, Figure 12e, and their dynamics less complex. The overarching result of the hybridization of mode 1 is the dominance of the response at the quarter-point and three quarters-point over the mid-point $w_{\max}\left(0.25,t\right)\;\&\;w_{\max}\left(0.75,t\right)>w_{\max}\left(0.5,t\right)$. Finally, comparison among the five cases shows that the primary resonance strengthens as the interaction between the modes becomes more active and is strongest on the lower side of the veering point, where the stiffness of mode 1 is relatively low, allowing for larger motions under a fixed excitation level.

## 5. Detection Mechanisms

We examine the effectiveness of four plausible detection mechanisms for asymmetric arch beam sensors.

### 5.1. Frequency Shift Detection

First, we examine the traditional frequency shift detection mechanism. This is based on the shift in the frequency of a resonant peak due to changes in the mass of the microbeam in the presence of the target gas. We used the eigenvalue problem to determine the responsivity of modes 1 and 2 to added mass as

$$R_{i}=\frac{\Delta \omega_{i}}{\Delta m}$$

for five values of the initial rise $w_{o}$ in the vicinity of the veering point by evaluating the change in the natural frequency of the relevant mode $\Delta \omega_{i}$ due to a finite but small added mass Δm. The results are listed in Table 5. Since the natural frequencies of the two modes are similar in this range, see Table 4 and Figure 9, a cursory inspection may suggest that their sensitivities would be similar. In fact, the sensitivity of the second mode is not impacted by changes in initial rise $w_{o}$ while the first mode sees significant changes. This is consistent with the sensitivity of inertial sensors derived by Ekinci et al. [42] as

(9) $$S=-\frac{2}{k_{e}m_{e}}$$

where $\delta m=S\delta \omega_{i}$ and $k_{e}$ and $m_{e}$ are the effective stiffness and mass of the mode. The asymmetric mode (mode 2) does not see changes in either mass or stiffness, while mode 1 sees a monotonic increase in effective stiffness as the initial rise increases.

### 5.2. Conventional Bifurcation-Based Detection

Bifurcation-based sensing mechanisms rely on a frequency shift a bifurcation point instead of a resonant peak and exploit the qualitative change in response beyond the bifurcation as a detector. We consider a sensor made of an arch microbeam with an initial rise of $w_{o}=6$ µm, where interactions between the modes are not active but mode 2 is hybridized. Inspecting the bifurcations identified in the sensor’s frequency–response curve, Figure 12e, we select two bifurcations, where the response jumps from a small stable orbit to a large stable orbit, namely CF_2_ and CF_6_, under a drop in the natural frequency, due to added mass, to implement the sensor. Those bifurcations provide for a reusable sensor since they avoid jumps to pull-in, and a large qualitative detection signal generated by a sudden expansion in the orbit size.

**Figure 13:**
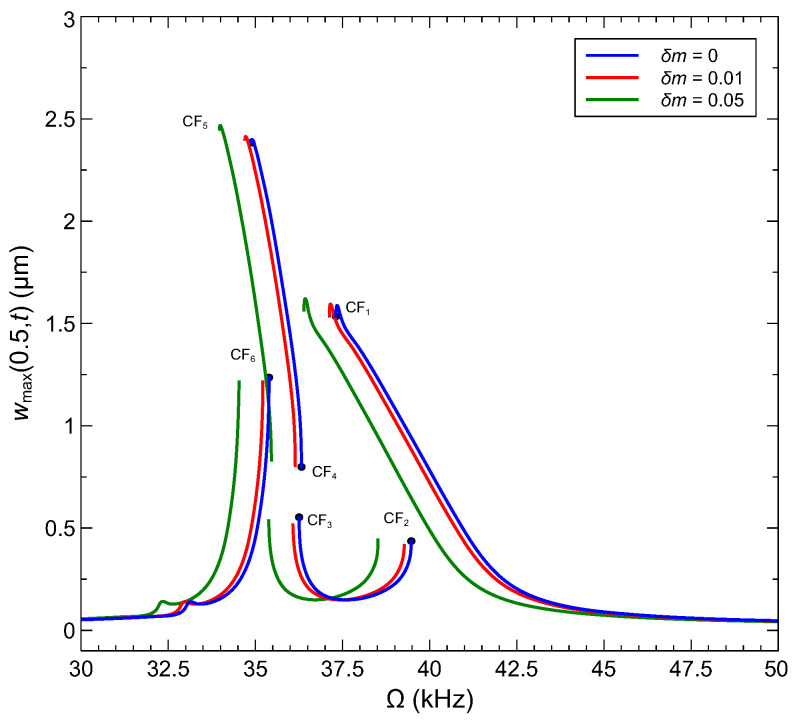
The frequency–response curves of bifurcation-based sensors based on arch microbeams with initial rise of $w_{o}=6$ µm under the voltage waveform $V_{DC}=V_{AC}=12.2$ V.

Figure 13 compares the frequency response of the sensor operating under the waveform $V_{DC}=V_{AC}=12.2$ V to its counterpart for two levels of added mass δm. In all three cases, only the stable branches of the frequency response are shown. The curves undergo a shift to the left with added mass, thereby shifting the location of each bifurcation point from $\omega_{\mathrm{CFi}}$ to a lower frequency ${\hat{\omega }}_{\mathrm{CFi}}$. As a result, the response of the sensor operating at the former bifurcation point $\omega_{\mathrm{CFi}}$ must jump to the upper branch. In Table 6, we report the responsivity of two sensors operating at $\Omega =\omega_{\mathrm{CF}2}=39.46$ kHz and $\Omega =\omega_{\mathrm{CF}6}=35.36$ kHz as

$$R_{i}=\frac{\Delta \omega_{\mathrm{CFi}}}{\Delta m}$$

where $\Delta \omega_{\mathrm{CFi}}=\omega_{\mathrm{CFi}}-{\hat{\omega }}_{\mathrm{CFi}}$. We find that the sensitivities of the bifurcation-based sensors are comparable to those of frequency shift sensors. The complex dynamics of the veering range offer opportunities to improve the responsivity of the former, while they undermine the responsivity of the latter or render them completely inoperable as complex dynamics turn regular (periodic) orbits at the resonant peak to quasiperiodic orbits, subsequent to a secondary Hopf bifurcation, or unstable orbits.

Conventional bifurcation-based sensors detect the presence or concentration of a gas by measuring a corresponding change in the orbit size subsequent to a jump from a lower to an upper branch of orbits. Capacitance-based transduction mechanisms are typically used for this purpose. The instantaneous capacitance of the sensor can be found in terms of the beam position with respect to the fixed electrode by integrating the capacitance per unit length, given by Equation (2), over the beam length

(10) $$\begin{array}{cc} C(t) & =\int_{0}^{L}C_{l}\;d\hat{x}=\int_{0}^{L}H_{c}\left(\hat{x}\right)\left(\frac{\varepsilon b}{d-\hat{w}+{\hat{w}}^{0}}-0.65\varepsilon \log\left(d-\hat{w}+{\hat{w}}^{0}\right)\right)\;d\hat{x}\\ \; & =\int_{0}^{\frac{1}{2}}\left(\frac{\varepsilon bL}{d(1-w+w^{0})}-0.65\varepsilon L\left(\log d+\log(1-w+w^{0})\right)\right)\;dx \end{array}$$

Since the measurand is the orbit size, rather than the instantaneous displacement, we evaluate the RMS of the capacitance over the orbit as a surrogate. We normalize the sensor capacitance with respect to its initial capacitance

$$C_{0}=\int_{0}^{\frac{1}{2}}\left(\frac{\varepsilon bL}{d(1+w^{0})}-0.65\varepsilon L(\log d+\log\left(1+w^{0}\right)\right)\;dx$$

and evaluate it as

(11) $$C_{\mathrm{RMS}}=\int_{0}^{\frac{2\pi }{\Omega }}\frac{C(t)}{C_{0}}\;dt$$

**Figure 14:**
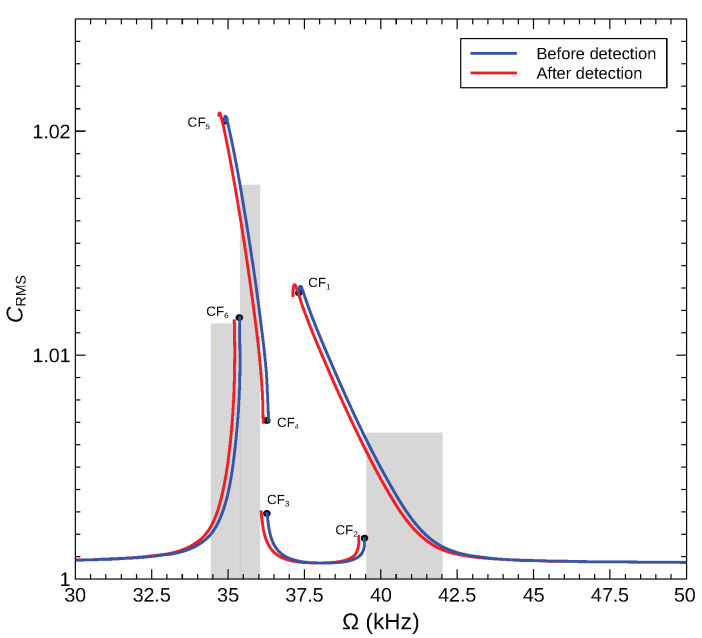
The normalized RMS capacitance for sensors made of an arch microbeam with an initial rise of $w_{o}=6$ µm under the voltage waveform $V_{DC}=V_{AC}=12.2$ V.

We show in Figure 14 the normalized RMS capacitance of an arch microbeam with an initial rise of $w_{o}=6$ µm under an excitation waveform of $V_{DC}=12.2=V_{AC}=12.2$ V in the frequency range of [30−50] kHz as well as their counterpart once an added mass of δm=0.01 is introduced. Two sensors based on the CF_2_ and CF_6_ bifurcations are examined. The sensors operate in the gray shaded regions of the figure with their excitation frequencies set to $\Omega =\omega_{\mathrm{CF}2}$ and $\Omega =\omega_{\mathrm{CF}6}$. The presence (or concentration) of the target gas results in the addition of a mass δm=0.01 and the sensor response jumping from the lower (blue colored) side of the bifurcation to the upper (red colored) side. The detection signal takes the form of an increase in the normalized RMS capacitance by 0.4%.

### 5.3. Modal Ratio Based Detection

We propose a novel detection method that exploits response asymmetry to amplify the detection signal (capacitance change) of bifurcation based sensors. The detector in this case is the ratio of the capacitance attributed to mode 1 to that attributed to mode 2 rather than the RMS of the total capacitance $C_{\mathrm{RMS}}$. This can be implemented by evaluating the FFT of the measured capacitance over multiple excitation periods and obtaining the ratio of the peaks corresponding to modes 1 and 2.

**Figure 15:**
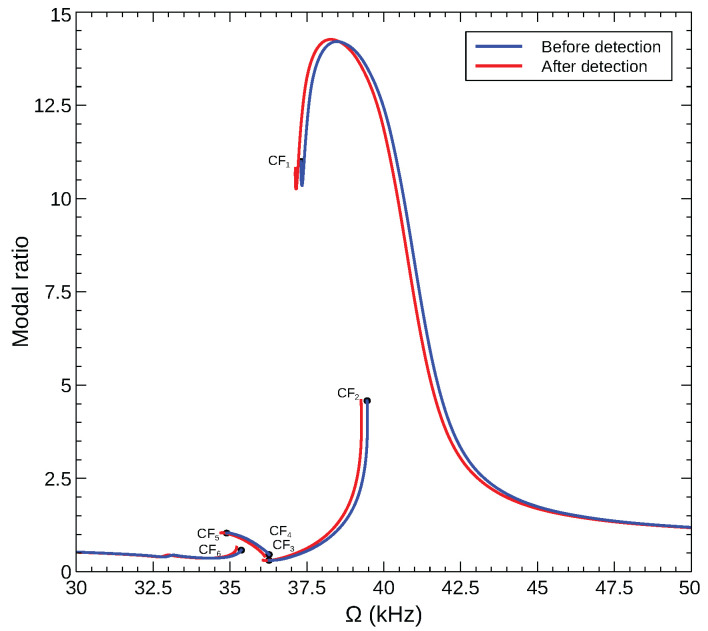
The amplitude ratio between the generalized coordinates of modes 1 and 2 $\left(P_{q_{1}}/P_{q_{2}}\right)$ for two sensors modes of an arched microbeam with an initial rise of $w_{o}=6$ µm under the voltage waveform $V_{DC}=V_{AC}=12.2$ V.

To demonstrate the feasibility of this detection method and to examine its efficacy, we calculated the corresponding ratio in the sensor model, Equation (A13), namely the ratio of the dominant peaks in the FFT of each of the generalized coordinates $\left(P_{q_{1}}/P_{q_{2}}\right)$. The modal amplitude ratio is shown in Figure 15 for the CF_2_ and CF_6_ sensors described above. The figure shows the modal ratios of the stable orbits before and after gas detection (added mass of δm=0.01). Upon gas detection, the modal ratio for sensor CF_2_ is found to increase from 4.6 to 13.2, whereas that of sensor CF_6_ increases from 0.6 to 0.9. Comparing the performance of the two sensors shows that the modal ratio can be a more sensitive measurand than the total capacitance, provided that it is used for sensors that undergo a significant change in the modal contributions across the operating bifurcation.

The use of a modal ratio detection mechanism would require the fabrication of three half-length electrodes placed, for example, north-east, south-east, and south-west of the arch beam. Under this configuration, the south-east electrode would be used for actuation. The weighted average of the current output of the south-east and south-west electrodes will be used to measure the response of the symmetric mode, while the weighted average of the north-east and south-west electrodes will be used to measure the response of the asymmetric mode. A similar configuration was implemented by Ahmed et al. [43].

### 5.4. Differential Capacitance Detection

**Figure 16:**
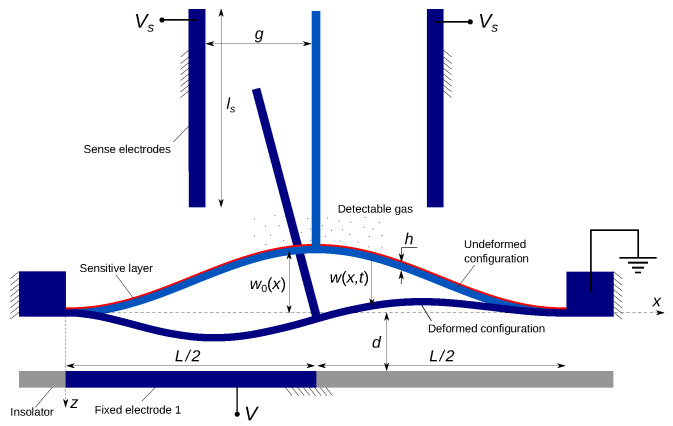
Schematic of the differential capacitance gas sensor.

The detection electronics of inertial MEMS gas sensors are challenging due to the presence of parasitics and, typically, low SNR. Many approaches have been proposed to realize practical readout circuits. For example, Park et al. [44] showed that the detection of the higher harmonics in the motion-induced current of the sensor can overcome those parasitics. We propose here a novel detection mechanism, based on the differential capacitive measurement, to mitigate the effects of parasitic capacitance. As such, we modify the sensor by incorporating a rigid arm extending transversely from the arch microbeam mid-point to enable the use of differential capacitive sensing. The arm is placed at an equal distance *g* from $l_{s}$-long right and left sense electrodes, Figure 16. The sensor is grounded, while the sense electrodes are held at a common DC voltage $V_{s}$. The detection signal, in this case, is the difference between the capacitance of the left capacitor $C_{sl}$ formed by the rigid arm and the left sense electrode and that of the right capacitor $C_{sr}$ formed by the rigid arm and the right sense electrode. In addition to rejecting common mode (parasitic) capacitance, this sensor design amplifies the signal generated by the rotary motions of the rigid arm induced by asymmetry in the sensor response. A similar design was proposed, fabricated, and successfully tested by Noori [45] as a vibratory MEMS gyroscope.

Bifurcation sensors designed using this detection mechanism will operate across bifurcations that trigger a sudden transition from nearly symmetric to strongly asymmetric beam motions and, therefore, large rotary motions of the rigid arm. Assuming that the rigid arm mass is negligible, the deflection and slope of the arch microbeam at the mid-point $w(\frac{1}{2},t)$ and $w^{\prime }\left(\frac{1}{2},t\right)$, respectively, obtained by integrating Equation (A13), can be used to calculate the instantaneous position of the rigid arm root point and its orientation with respect to the median line as

(12) $$\begin{array}{cc} w_{m}\left(t\right) & =w(\frac{1}{2},t) \end{array}$$

(13) $$\begin{array}{cc} \theta (t) & =w^{\prime }\left(\frac{1}{2},t\right) \end{array}$$

**Figure 17:**
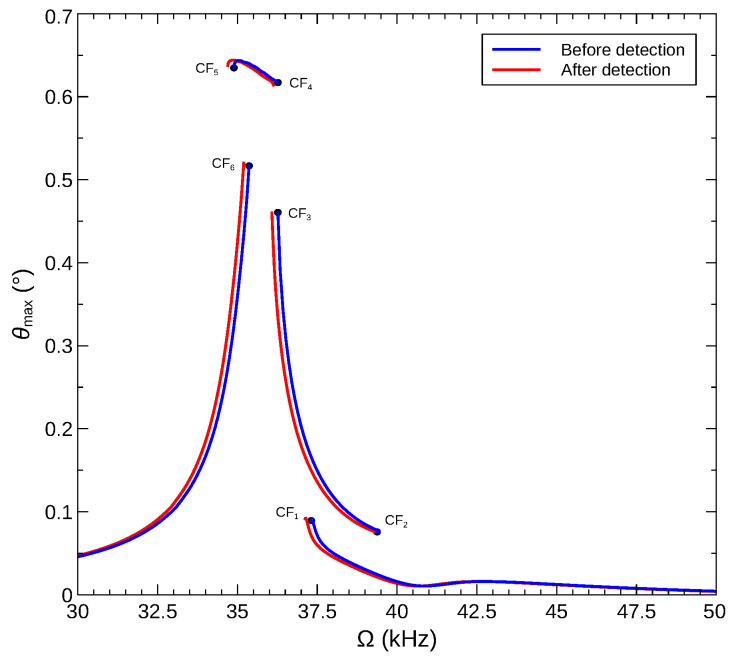
The maximum rotation angle of the rigid arm over the orbit for sensors made of an arch microbeam with an initial rise of $w_{o}=6$ µm under the voltage waveform $V_{DC}=V_{AC}=12.2$ V.

Figure 17 shows the maximum rotation angle of a rigid arm $\theta_{\max}$ attached to the arch microbeam actuated with voltage waveform described in Section 5.2. Comparing the frequency–response curves before and after gas detection (addition of a mass of δm=0.01) reveals that a sensor operating across the CF_6_ bifurcation $\left(\Omega =\omega_{\mathrm{CF}6}\right)$ will see a significant jump in rotational motions of the arm upon gas detection. On the other hand, a sensor operating across the CF_2_ bifurcation $\left(\Omega =\omega_{\mathrm{CF}2}\right)$ will see an undesirable drop in rotational motions.

We define the normalized differential capacitance as

(14) $$C_{\mathrm{diff}}\left(t\right)=\frac{C_{sl}\left(t\right)-C_{sr}\left(t\right)}{C_{so}}$$

where $C_{so}=\frac{\varepsilon bl_{s}}{g}$. The capacitance of the right and left sense capacitors for small rotation angles, such as those observed above, can be written as

(15) $$\begin{array}{cc} \; & C_{sr}\left(t\right)=\int_{0}^{l_{s}-dw_{m}}\frac{\varepsilon b}{g-\frac{d}{L}\theta \xi }d\xi =\frac{\varepsilon b}{\frac{d}{L}\theta }\left(\log g-\log\left(g-\frac{d}{L}\theta \left(l_{s}-dw_{m}\right)\right)\right) \end{array}$$

(16) $$\begin{array}{cc} \; & C_{sl}\left(t\right)=\int_{0}^{l_{s}-dw_{m}}\frac{\varepsilon b}{g+\frac{d}{L}\theta \xi }d\xi =\frac{\varepsilon b}{\frac{d}{L}\theta }\left(-\log g+\log\left(g+\frac{d}{L}\theta \left(l_{s}-dw_{m}\right)\right)\right) \end{array}$$

Setting the length of the rigid arm and the right and left sense electrodes to $l_{s}=650$ µm and the gap distance between them at rest to g=9 µm, we calculate the differential capacitance.

**Figure 18:**
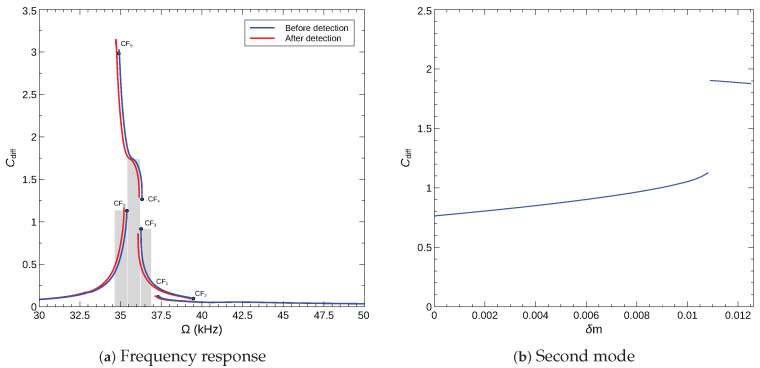
Differential capacitance detection mechanism: (**a**) the maximum normalized differential capacitance over the orbit for sensors made of an arch microbeam with an initial rise of $w_{o}=6$ µm under the voltage waveform $V_{DC}=V_{AC}=12.2$ V, (**b**) the calibration curve of the bifurcation based sensor operating across CF_6_ with an excitation frequency of Ω=35.2 kHz.

We used Equation (14) to evaluate the maximum of the normalized differential capacitance over the orbit for the arch microbeam and the voltage waveform described in Section 5.2. The frequency–response curves before and after gas detection (addition of a mass of δm=0.01) are shown in Figure 18a. As expected, differential capacitance proves to be an effective measurand for the sensor operating across the CF_6_ bifurcation, with $\Omega =\omega_{\mathrm{CF}6}$, but a poor measurand for a sensor operating across the CF_2_ bifurcation $\left(\Omega =\omega_{\mathrm{CF}2}\right)$ where differential capacitance drops upon detection. We observe that using this measurand, an effective bifurcation sensor can also be designed to operate across the CF_3_ bifurcation.

We plot the calibration curve of a sensor with an operating frequency of Ω=35.2 kHz in Figure 18a. It shows that using the maximum normalized differential capacitance $C_{\mathrm{diff}}$ as a measurand, the sensor can operate in both analog and binary modes. The sensor operates in analog mode up to an added mass of δm=0.0108 with a nearly linear calibration curve. In binary mode, the detection jump (subsequent to addition of δm=0.0109) results in a 30% increase in differential capacitance.

To estimate the sensor’s limit of detection (LoD) under this detection mechanism capacitance, we set the minimum detectable capacitance to $C_{min}=0.003$ pF corresponding to that of the Agilent U1733C LCR Meter. The corresponding normalized differential capacitance can be calculated using Equation (14) as ${\left(C_{\mathrm{diff}}\right)}_{min}=0.156$. Using the calibration curve shown in Figure 18b, it corresponds to δm=0.0066 and then results in an LoD of 0.66% of the beam mass.

## 6. Conclusions

This work explores the rich possibilities of electrostatically actuated arch microbeams present for the design of inertial gas sensors. In particular, it focuses on exploiting the interaction between symmetric and asymmetric modes to create various detection mechanisms and compares their sensitivity. The sensor is actuated via a half-length electrode to activate both symmetric and asymmetric modes. A dynamic reduced-order model of the sensor was developed and validated, then used to investigate the sensor response.

Our findings show that under the traditional frequency shift detection mechanism, veering does not affect the responsivity, defined as the ratio of the change in the frequency to the added mass, of asymmetric modes. On the other hand, it slightly increases the responsivity of symmetric modes, as the increase in initial rise, that drives veering, also increases the effective stiffness. Therefore, a lower initial rise that does not approach veering is recommended for this detection mechanism.

Comparison between frequency shift and bifurcation-based sensors operating under similar conditions shows that they have comparable sensitivities. On the other hand, as sensor motions grow larger, whether due to increased excitation levels or the availability of stronger resonant conditions (such as those observed in the veering range), bifurcation-based sensors can realize better sensitivity and/or higher SNR, whereas frequency shift sensors become inoperable as complex dynamics turn the regular (periodic) orbits present at smaller resonant peaks to quasiperiodic orbits, chaotic attractors, or unstable orbits at larger resonant peaks.

The paper examined two novel gas detection mechanisms. The first uses the modal participation ratio of an arch beam. The second adds a transverse arm and uses differential capacitance measurements. The modal interactions within the veering range as well as the mode hybridization realized beyond that range offer opportunities to improve the sensitivity and SNR of bifurcation-based sensors. We demonstrated that a modal ratio-based detection method increases the size of the detection signal by three orders of magnitude from that of conventional bifurcation-based sensors. Another method to exploit those rich dynamics is to use the rotational motions induced by asymmetries in the sensor response to realize differential capacitance measurements. The latter was found to increase the signal level by by two orders of magnitude from that of conventional bifurcation-based sensors.

Those detection methods allow bifurcation sensors to replace conventional capacitance-based transduction mechanisms with mechanisms that offer larger SNR. They also allow the sensors to operate in analog or binary modes. However, this class of detection methods need to be tailored to the specific modal interactions underlying the bifurcation; otherwise, they may degrade the SNR rather than improve it.

Finally, we note that curved beam resonators require the use of a thick structural layer, which limits available fabrication processes to SOI and similar processes. It also poses challenges in the characterization of those sensors since classical vibrometers cannot detect in-plane motion, and electric measurements cannot differentiate between modes involved in 1:1 interactions since they have the same frequency-domain profile.

## Figures and Tables

**Table 1:** Dimensions and material properties of the microbeam used for model validation.

*L* (μm)	*b* (μm)	*h* (μm)	*d* (μm)	*E* (GPa)	ρ (kg/m${}^{3}$)
400	15	1.9	1.8	166	2330

**Table 2:** Measured vs. calculated natural frequencies of the microbeam subject to half-electrode actuation ($V_{DC}=2.45$ V).

Natural Frequency (kHz)	$\omega_{1}$	$\omega_{2}$	$\omega_{3}$
Experiments	45.53	206.44	450.78
Model	44.39	220.64	490.99

**Table 3:** Dimensions and material properties of the sensor.

*L* (μm)	*b* (μm)	*h* (μm)	*d* (μm)	*E* (GPa)	ρ (kg/m${}^{3}$)
1000	30	1.7	9	130	2332

**Table 4:** The natural frequencies of the arch microbeam actuated with a half-electrode and $V_{DC}=15$ V.

Initial Rise	Mode 1 (kHz)	Mode 2 (kHz)	Mode 3 (kHz)
$w_{o}=3$ µm	27.56	35.27	76.26
$w_{o}=4$ µm	33.15	35.53	81.98
$w_{o}=4.5$ µm	35.06	35.98	85.40
$w_{o}=5$ µm	37.57	35.46	89.15
$w_{o}=6$ µm	40.52	35.61	97.41

**Table 5:** Responsivity of arch microbeam sensors with an initial rise of $w_{o}=6$ µm based on the shift in the resonant peak of modes 1 and 2 and operating under a waveform with RMS voltage of $V_{\mathrm{RMS}}=15$ V.

Initial Rise $w_{o}$	3	4	4.5	5	6
Mode 1 sensor (Hz/pg)	0.113	0.136	0.145	0.155	0.167
Mode 2 sensor (Hz/pg)	0.145	0.147	0.148	0.146	0.147

**Table 6:** Responsivity of bifurcation-based arch microbeam sensors with an initial rise of $w_{o}=6$ µm operating under the voltage waveform $V_{DC}=12.2=V_{AC}=12.2$ V with an excitation frequency $\Omega =\omega_{\mathrm{CF}2}$ and $\Omega =\omega_{\mathrm{CF}6}$.

δm	0.01	0.05
CF_2_ sensor (Hz/pg)	0.165	0.16
CF_6_ sensor (Hz/pg)	0.148	0.144

## Data Availability

Data available from the corresponding author on reasonable request.

## Appendix A. Static Reduced-Order Model

The static reduced-order model is formulated by applying DQM on the static equation. We obtain the following set of algebraic equations:

(A1) $$\begin{array}{cc} \left(1+\frac{\alpha_{1}}{B_{p}}\right) & \sum_{j=1}^{n}A_{i,j}^{\left(4\right)}w_{j}^{s}-\alpha_{2}\left(\sum_{j=1}^{n}A_{ij}^{\left(2\right)}w_{j}^{s}-{w_{i}^{0}}^{\prime \prime }\right)\\ \times & \sum_{k=1}^{n}B_{k}\left[{\left(\sum_{j=1}^{n}A_{kj}^{\left(1\right)}w_{j}^{s}\right)}^{2}-2\sum_{j=1}^{n}A_{kj}^{\left(1\right)}w_{j}^{s}{w_{k}^{0}}^{\prime }\right]\\ + & \alpha_{3}V_{DC}^{2}H_{c}\left(x_{i}\right)\frac{1+\gamma (1-w_{i}^{s}+w_{i}^{0})}{{\left(1-w_{i}^{s}+w_{i}^{0}\right)}^{2}}=0\\ \; & i=3,\cdots ,n-2 \end{array}$$

and the boundary conditions

(A2) $$\begin{array}{c} w_{1}^{s}=w_{n}^{s}=0,\;\;\;\;\sum_{j=1}^{n}A_{1j}^{\left(1\right)}w_{j}^{s}=\sum_{j=1}^{n}A_{nj}^{\left(1\right)}w_{j}^{s}=0 \end{array}$$

where the nodal static solution are denoted by $w_{i}^{s}=w\left(x_{i}\right)$ and $w_{i}^{0}=w^{0}\left(x_{i}\right)$ and the DQM grid points are defined as

$$x_{i}=\frac{1}{2}\left[1-\cos\left(\frac{i-1}{n-1}\pi \right)\right]\;i=1,\cdots ,n$$

and $A_{ij}^{\left(p\right)}$ is the coefficient of the $p^{th}$-order derivative. These coefficients are given, here for the *x*-direction, by the following recursive formulas:

(A3) $$\begin{array}{cc} \; & A_{ij}^{\left(1\right)}=\frac{\prod_{v=1;v\neq i}^{n}\left(x_{i}-x_{v}\right)}{\left(x_{i}-x_{j}\right)\prod_{v=1;v\neq j}^{n}\left(x_{j}-x_{v}\right)}\\ \; & A_{ij}^{\left(p\right)}=p\left(A_{ii}^{p-1}A_{ij}^{1}-\frac{A_{ij}^{p-1}}{\left(x_{i}-x_{j}\right)}\right)\\ \; & A_{ii}^{\left(p\right)}=-\sum_{v=1;v\neq i}^{n}A_{iv}^{\left(p\right)}\\ \; & \;i,j=1,2,\cdots ,n \end{array}$$

The Newton–Cotes coefficients, used for the discretization of the integral term, are given by

(A4) $$\begin{array}{c} B_{i}=\int_{0}^{1}\prod_{j=1;j\neq i}^{n}\frac{x-x_{j}}{x_{i}-x_{j}}dx\;\;\;\;\;i=1,2,\cdots ,n \end{array}$$

## Appendix B. Damped Eigenvalue Problem

The damped eigenvalue problem is obtained by considering a dynamic solution expressed as

(A5) $$w\left(x,t\right)=w^{s}\left(x\right)+\phi^{r}\left(x\right)e^{i\lambda_{r}t}$$

where the superscript *s* denotes the static position. We substitute Equation (A5) into Equation (3), linearize the outcome around the static position $w^{s}$, and apply the DQM to obtain a set of linear algebraic equations. The obtained equations can be written in matrix form as

(A6) $$\begin{array}{c} \left[-\lambda_{r}^{2}M+i\lambda_{r}C+K\left(w^{s}\right)\right]X=0 \end{array}$$

where

(A7) $$\begin{array}{c} X^{T}=\left(\phi_{3}^{r}\;\phi_{4}^{r}\cdots \phi_{n-2}^{r}\right) \end{array}$$

and

(A8) $$\begin{array}{c} {\left(w^{s}\right)}^{T}=\left(w_{3}^{s}\;w_{4}^{s}\cdots w_{n-2}^{s}\right) \end{array}$$

Equation (A6) is rewritten to be solved as an algebraic eigenvalue problem [46]:

(A9) $$BY=\lambda_{r}AY$$

where

$$B=\left[\begin{array}{cc} 0 & I\\ K & C \end{array}\right]\mathrm{and}A=\left[\begin{array}{cc} I & 0\\ 0 & M \end{array}\right]$$

and $Y^{T}=\left(X\;\lambda_{r}X\right)$.

Equation (A9) is solved to obtain the eigenvalues along with the eigenvectors (bending and pressure mode shapes evaluated at the DQM grid points). We define the damped natural frequency, the damping ratio and quality factor for mode *i*, respectively, as follows:

(A10) $$\omega_{i}=\left|\lambda_{i}\right|\sqrt{1-\xi_{i}^{2}},\;\xi_{i}=\frac{-Re(\lambda_{i})}{\left|\lambda_{i}\right|},\;Q_{i}=\frac{1}{2\xi_{i}}$$

## Appendix C. Dynamic Reduced-Order Model

The nonlinear dynamic reduced-order model is obtained by applying the Galerkin approach for space discretization. The transverse deflection of the microbeam w(x,t) is expressed as

(A11) $$\begin{array}{cc} \; & w\left(x,t\right)=\sum_{j=1}^{m}q_{j}\left(t\right)\phi^{j}\left(x\right) \end{array}$$

where the spatial function $\phi^{i}\left(x\right)$ is the $i^{th}$- linear normalized nondimensional undamped mode shape of a clamped-clamped beam and the time-varying function $q_{i}\left(t\right)$ is the corresponding modal coordinate. *m* denotes the number of the retained modes. The nondimensional form of the mode shapes is given by

(A12) $$\phi \left(x\right)=\cosh\left(\beta x\right)-\cos\left(-\beta x\right)+\frac{\cos(-\beta )-\cosh(\beta )}{\sinh(\beta )+\sin(-\beta )}\left(\sinh(\beta x)+\sin(-\beta x)\right)$$

where β is solution of 1−cosβcoshβ=0.

We substitute Equation (A11) into Equation (3), multiply the outcome by the mode shape $\phi^{i}$, and integrate the resulting equations from 0 to 1. We apply DQM and Newton–Cotes to approximate the unknown integrals and spatial derivatives at the DQM grid points. We obtain the following equations that constitute the nonlinear reduced-order model of the asymmetric arch beam sensor:

(A13) $$\begin{array}{cc} {\ddot{q}}_{i}+ & \left(\mu_{i}+\sum_{k=1}^{n}B_{k}\phi_{k}^{i}\frac{\mu_{eff}}{{\left(1-\sum_{j=1}^{m}q_{j}\phi_{k}^{j}+w_{k}^{0}\right)}^{3}}\right){\dot{q}}_{i}+\beta_{i}^{4}q_{i}+\alpha_{1}\phi_{p}^{i}\sum_{j=1}^{m}q_{j}\phi_{p}^{j}\\ \; & =\alpha_{2}\left(\sum_{j=1}^{m}q_{j}\int_{0}^{1}\phi^{i}\phi_{,xx}^{j}\;dx-\int_{0}^{1}\phi^{i}w_{,xx}^{0}\;dx\right)\\ \times & \sum_{j=1}^{m}\left(q_{j}^{2}\int_{0}^{1}{\phi_{,x}^{j}}^{2}\;dx+2\sum_{k=1}^{j-1}q_{j}q_{k}\int_{0}^{1}\phi_{,x}^{j}\phi_{,x}^{k}\;dx-2q_{j}\int_{0}^{1}\phi_{,x}^{j}{w^{0}}^{\prime }\;dx\right)\\ + & \alpha_{3}V^{2}H_{c}\left(x_{i}\right)\sum_{k=1}^{n}B_{k}\phi_{k}^{i}\frac{1+\gamma \left(1-\sum_{j=1}^{m}q_{j}\phi_{k}^{j}+w_{k}^{0}\right)}{{\left(1-\sum_{j=1}^{m}q_{j}\phi_{k}^{j}+w_{k}^{0}\right)}^{2}}\\ \; & i=1,\cdots ,m \end{array}$$

where $\phi_{k}^{i}=\phi^{i}\left(x_{k}\right)$ and $w_{k}^{0}=w^{0}\left(x_{k}\right)$.
